# Electrochemical Aptasensor for Endocrine Disrupting 17β-Estradiol Based on a Poly(3,4-ethylenedioxylthiopene)-Gold Nanocomposite Platform

**DOI:** 10.3390/s101109872

**Published:** 2010-11-03

**Authors:** Rasaq A. Olowu, Omotayo Arotiba, Stephen N. Mailu, Tesfaye T. Waryo, Priscilla Baker, Emmanuel Iwuoha

**Affiliations:** Sensor Lab, Department of Chemistry, University of the Western Cape, Bellville, 7535, South Africa; E-Mails: rolowu@uwc.ac.za (R.A.O.); oarotiba@uwc.ac.za (O.A.); 2970836@uwc.ac.za (S.N.M.); twaryo@uwc.ac.za (T.T.W.); eiwuoha@uwc.ac.za (E.I.)

**Keywords:** poly(3,4-ethylenedioxythiophene) (PEDOT), aptamer, modified electrode, avidin/biotin, square wave voltammetry

## Abstract

A simple and highly sensitive electrochemical DNA aptasensor with high affinity for endocrine disrupting 17β-estradiol, was developed. Poly(3,4-ethylenedioxylthiophene) (PEDOT) doped with gold nanoparticles (AuNPs) was electrochemically synthesized and employed for the immobilization of biotinylated aptamer towards the detection of the target. The diffusion coefficient of the nanocomposite was 6.50 × 10^−7^ cm^2^ s^−1^, which showed that the nanocomposite was highly conducting. Electrochemical impedance investigation also revealed the catalytic properties of the nanocomposite with an exchange current value of 2.16 × 10^−4^ A, compared to 2.14 × 10^−5^ A obtained for the bare electrode. Streptavidin was covalently attached to the platform using carbodiimide chemistry and the aptamer immobilized via streptavidin—biotin interaction. The electrochemical signal generated from the aptamer–target molecule interaction was monitored electrochemically using cyclic voltammetry and square wave voltammetry in the presence of [Fe(CN)_6_]^−3/−4^ as a redox probe. The signal observed shows a current decrease due to interference of the bound 17β-estradiol. The current drop was proportional to the concentration of 17β-estradiol. The PEDOT/AuNP platform exhibited high electroactivity, with increased peak current. The platform was found suitable for the immobilization of the DNAaptamer. The aptasensor was able to distinguish 17β-estradiol from structurally similar endocrine disrupting chemicals denoting its specificity to 17β-estradiol. The detectable concentration range of the 17β-estradiol was 0.1 nM–100 nM, with a detection limit of 0.02 nM.

## 1. Introduction

Within the last ten years, many research articles describing modified electrodes using nanoparticles and conducting polymers have been published [1–4]. The field of electrochemical biosensors has grown rapidly because they provide fast, simple and inexpensive detection capabilities for biological binding events [5]. Conducting polymers can be obtained either chemically or electrochemically from their corresponding monomers, but the electropolymerization technique is more versatile. The film thickness and characteristics can be controlled by monitoring the polymerization charge during electropolymerization [6]. Conducting polymers exhibit unique properties such as catalysis, conductivity, biocompatibility, as well as their ability to act as an electrical plug connecting the bio recognition element to the surface of the electrode [7–9].

In order to improve sensitivity, selectivity and stability of biosensors different kinds of nanocomposite materials with enhanced performance have been developed and utilized for biosensor fabrication [10–12]. Nanocomposites containing inorganic nanoparticle and conducting polymers show a facile flow of electronic charge and unique properties that are obtained with these materials [2,13]. Nanocomposite materials exhibit exceptional electrical properties and optical properties compared to conducting polymer or metal nanoparticles used individually [13,14]. The electrocatalytic properties of nanoparticles are enhanced by the favorable environment supplied by the polymeric matrix [15]. The composite of conducting polymer-metal nanoparticle can be obtained from different metals and π-conjugated polymers as well as oligomer linkers which have received considerable attention due to the possibilities of creating suitable materials for electrocatalysis, chemical sensor and microelectronics [14,15]. Nanocomposite materials involving a hybrid of gold nanoparticle and carbon nanotube have been utilized for the construction of DNA biosensors [16]. Feng and his co-workers reported the use of a nanocomposite material consisting of gold nanoparticle and polyaniline nanotube membrane for DNA biosensor which was employed as a platform for immobilization of the DNA probe [2].

Among the various conducting polymers and conducting polymer nanocomposite materials developed and studied over the past ten years, poly(3,4-ethylenedioxythiopene) (PEDOT)-metal nanocomposites have been the focus of several studies as a result of its unique properties such as excellent stability against biological reducing agent due to their more ordered structure, high conductivity, high transparency and good thermal stability [17–19]. These unique properties make PEDOT an excellent material for various applications such as electrochromics, sensor light-emitting diodes and antistatic coatings [20,21]. The superior electrochemical stability of PEDOT may be attributed to the presence of the ethylenedioxy binding group on the α and β position of thiophene ring in EDOT, which blocks coupling along the backbone, making the resulting polymer regiochemically defined [21]. PEDOT has been reported to be excellent for synthesis of nanostructured materials and devices as a result of their electrical, electronic, magnetic, and optical properties which is similar to metals or semiconductors [22,23]. PEDOT coatings can be prepared by electrochemical polymerization in aqueous solution which allows the direct incorporation of water soluble anions [7,24]. Xiao and co-workers have developed an adenosine 5′-triphosphate (ATP) doped PEDOT for neural recording [25]. A silver nanograin incorporating a poly(3,4-ethylenedioxythiophene) (PEDOT) modified electrode for electrocatalytic sensing of hydrogen peroxide by simple electrochemical method was reported recently by Balamurugan and his co-workers [26].

One of the major classes of environmental contaminants are the endocrine disrupting chemicals (EDCs), which interfere with the function of the endocrine system. In recent years, these endocrine disrupting chemicals have become one of the main topics of research in the field of environmental sciences [27]. EDCs are defined as exogenous substances that cause adverse effect in organisms or its progeny, consequent to changes in endocrine function. They are ubiquitous in the environment because of their large number of uses in the residential, industrial and agricultural application [28]. The detection of these chemicals within the natural system is therefore necessary for protecting public and environmental health. Instrumental analysis methods such as high performance liquid chromatography (HPLC) and gas chromatography coupled with mass spectrophotometer (GC/MS) are very sensitive at detecting these toxic endocrine disrupting chemicals but are also very complicated to perform and require long analysis times to be accomplished. Using GC/MS an estimated concentration of 0.18–19.1 μg/kg was obtained in different foodstuffs in Germany compared to a calculated daily intake of 7.5 μg/day for nonylphenol, an endocrine disrupting chemical [29]. Concentrations of between 7.7–11,300 ng/L for some phenolic endocrine disrupting chemicals were obtained from river water in China using GC/MS coupled with the negative chemical ionization (NCI) technique [30]. The estimated daily human intake of estrogenic and anti-estrogenic counterparts, based on *in vitro* potencies relative to 17β-estradiol, has indicated that a woman taking birth control pills ingests about 6,675 μg/equivalent per day, postmenopausal estrogen treatment amount to 3,350 μg and ingestion of estrogen flavonoids in food represent 102 μg, while the daily ingestion of environmental organochlorine estrogen was estimated to be 2.5 × 10^−6^ μg [31].

Since the mid 1990s, a variety of adverse effects of endocrine disrupting chemicals on the endocrine systems of man and animals have been observed, which are of particular environmental concern [32]. Most EDCs are synthetic organic chemical that are introduced to the environment by anthropogenic sources but they can also be naturally generated by the estrogenic hormones 17β-estradiol and estrone [28]. It has been stated that the increasing incidence of breast and testicular cancers in humans may be caused by exposure to EDCs especially via drinking water whose source are often from surface waters [33,34]. The fate and behavior of EDCs are influenced by the structure and physicochemical parameters [35]. Most of these chemicals are present at low concentrations but many of them raised considerable toxicological concern. Children born to women exposed to high levels of polychlorobiphenyl (PCB) via consumption of polluted fish oil or rice oil have been reported to show delayed mental growth with lower Intelligence Quotient (IQ) scores, cognitive dysfunction, poorer visual identification memory and behavioral difficulties [36]. The incident of testicular cancer in men has increased significantly during the last decade. The incident of cancer in men under 50 years of age has increased approximately to 2–4% per annum since the 1960s in Great Britain, while in Denmark the most common malignancy among men from age 25–34 years is testicular cancer. Breast cancer is the most common tumor in women in the World. The relative rate of recurrence varies five-fold between countries with the highest incidence in Western Europe and in North America. There has been a steady increase of breast cancer incidence rates over the last decades everywhere in Europe. The increased risk in of cancer has been linked to exposure to estrogenic chemicals and it has been reported that woman exposed to organochlorine chemicals such as DDT and certain PCB congeners may have higher incidence of breast cancer than non-exposed woman [36]. Prostate cancer is the second leading form of cancer in males in USA. Deaths due to prostate cancer have increased by 17% over the past three decades, despite improved diagnosis [36]. Considering the serious adverse effect of EDCs on human health and environment even at low concentration, it is urgent to identify an efficient method of estimating the concentration level of these chemicals in the environment.

To assess the impact of these chemical in the environment requires improved analytical methods and tools [37]. Recently more attention has been focused toward electrochemical aptasensor based on modified electrode for the determination of environmental pollutants. This is due to their excellent analytical performance such as specificity, selectivity, simplicity, wide linear range response, reproducibility and low cost [38,39].

In this work we report the electrochemical synthesis of poly(3,4-ethylenedioxylthiopene)-PEDOT doped with gold nanoparticles (AuNPs) for the immobilization of biotinylated aptamer for the detection of 17β-estradiol—an endocrine disruptor. The single strand DNA which is the aptamer was chosen from that which has been reported in literature. This means that the sequence has been selected and patented and thus available for use. The sequence was designed according to literature [40]. The aptamer was biotinylated so as to effect a better immobilization chemistry using streptavidin—biotin interaction. The nanocomposite platform was characterized electrochemically by cyclic voltammetry in the presence of a [Fe (CN)_6_]^−3/−4^ redox probe.

## 2. Experimental Section

### 2.1. Chemicals and Reagents

3,4-Ethylenedioxythiophene (EDOT), 3,3-dithiodipropionic acid (DPA), *N*-(3-dimethylaminopropyl)-*N*-ethylcarbodiimide hydrochloride (EDAC), *N*-hydroxysuccinimide (NHS), streptavidin, HAuCl_4_·3H_2_O, 17β-estradiol, sodium monohydrogen phosphate, potassium dihydrogen phosphate, lithium perchlorate, potassium ferricyanide [K_3_Fe(CN)_6_] and potassium ferricyanide [K_4_Fe(CN)_6_] and sodium dodecylsulphate were procured from Sigma-Aldrich (South Africa). All chemicals were of analytical grade and were used as received. Deionized water (18.2 MΩ) purified by a milli-Q™ system (Millipore) was used throughout the experiment for aqueous solution preparation. A 76-mers biotinylated ssDNA aptamer synthesized by Inqaba Biotechnical Industries (Pty) Ltd., Hatfield, South Africa was used as aptamer probe. The sequence of the 76-mers sized biotinylated aptamer is given below:

**5′-BiotinGCTTCCAGCTTATTGAATTACACGCAGAGGGTAGCGGCTCTGCGCATTCAATTGCTGCGCGCTGAAGCGCGGAAGC-3′**

### 2.2. Solutions

Phosphate buffer saline (PBS) of pH 7.5 containing 10 mM Na_2_HPO_4_, KH_2_PO_4_ and 0.1 M KCl was prepared, 5 mM (1:1) solution of K_3_Fe(CN)_6_ and K_4_Fe(CN)_6_ was prepared in 100 mL of PBS at pH 7.5. One hundred μM biotinylated DNA aptamer stock was prepared in tris EDTA (TE) buffer (pH 8.0) and stored at −20 °C. Working DNA aptamer solution were prepared by diluting to the desired concentration in phosphate buffer storage at 4 °C and discarded after four weeks. Ten mL solution of 0.1 M lithium perchlorate containing 0.1 M EDOT monomer and 0.1 M of sodium dodecyl sulphate were prepared for the electropolymerization. A stock solution of 50 mL of binding buffer (pH 8.0) containing 100 mM tris HCl, 200 mM NaCl, 25 mM KCl ,10 mM MgCl_2_ and 5% ethanol were prepared. Stock solution of 1 mM *N*-[3-(dimethylaminopropyl)-*N*-3-ethylcarbodiimide hydrochloride] (EDAC), 1 mM *N*-hydroxysuccinimide (NHS), 1 mM 3,3-dithiodipropionic acid (DPA), as well as stock solution of 50 μg/mL septravidin in PBS (pH 7.5) were prepared. One hundred μM stock solution of 17β-estradiol (target) solution was prepared from which working target solution was prepared and diluted to desired concentrations with binding buffer. All stock solutions prepared were stored at 4 °C before and after use.

### 2.3. Electrochemical Measurement

A three electrode system was used to perform all electrochemical experiments. A gold electrode with a diameter of 1.6 mm was used as the working electrode, platinum wire as the counter electrode, and Ag/AgCl (3M Cl^−^) as the reference electrode. All electrochemical (voltammetric) experiments were recorded with Zahnner IM6 electrochemical workstation (MeBtechnik).

Square wave voltammetry (SWV) measurements were recorded at amplitude 25 mV and frequency of 15 Hz with this electrochemical work station. Cyclic voltammetry measurements were recorded with the same electrochemical workstation. All solutions were de-aerated by purging with argon through it for 5 mins. The experiments were carried out under room temperature. UV/Vis spectra measurements were recorded with the Nicolette Evolution 100 Spectrometer (Thermo Electron Corporation, UK). Transmission electron microscopy (TEM) was performed in a Tecnai G^2^ F_2_O X-Twin MAT. TEM characterizations were performed by placing a drop of the solution on a carbon coated copper grid and dried under electric UV lamp for 30 minutes.

### 2.4. Preparation of Gold Nanoparticles

AuNP was prepared by citrate reduction of HAuCl_4_ solution according to literature [13,41]. The AuNP prepared was stored in a brown bottle at 4 °C before use.

### 2.5. Preparation of a Poly(3,4-ethylenedioxythiopene) Modified Gold Electrode

Prior to surface modification, the gold working electrode was polished to a mirror-like surface with alumina powder of sizes 1.0, 0.3 and 0.05 μm respectively. The electrode was dipped in piranha solution for 10 minutes for effective cleaning and the electrode was then sonicated in ethanol and water consecutively for 5 mins. The electrode was further cleaned electrochemically in sulphuric acid by cycling between the potential of −200 mV to 1,500 mV until a reproducible cyclic voltammogram was obtained. Subsequently the gold electrode was rinsed with copious amount of water and absolute ethanol respectively. PEDOT film was deposited on the bare gold electrode from an aqueous solution of 0.1 M lithium perchlorate containing 0.1 M 3,4-ethylenedioxylthiopene and 0.1 M sodium dodecyl sulphate by cycling the potential between −1,000 mV to 1,000 mV at a scan rate of 50 mV/s for ten cycles, as shown in Figure 2.

### 2.6. Fabrication of PEDOT—Gold Nanoparticle Modified Electrode

The PEDOT modified gold electrode prepared above was immersed in a colloidal gold nanoparticles solution for 24 h for self assembly of the gold nanoparticles on the PEDOT film to obtain a nanocomposite modified electrode.

### 2.7. Fabrication of the Aptasensor

**Scheme 1.**
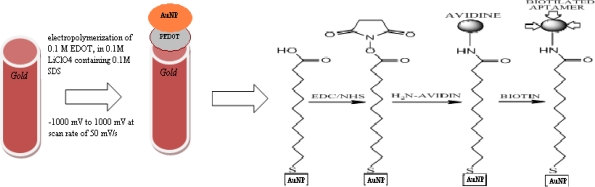
The schematic representation of the immobilization of aptamer on nanocomposite.

A self assembled monolayer of 3,3′-dithiodipropionic acid (DPA) on the PEDOT/AuNP modified electrode was formed by incubation in an ethanolic solution of 3 mM dithiodipropionic acid for 45 min. The unbound DPA was removed by washing the electrode in absolute ethanol and then Millipore water respectively. After that the self assembled DPA was activated by immersing the electrode in the same volume of 1 mM each of EDAC and NHS to activate the carboxylic group for easy bonding with the amine group of streptavidin for 90 min. The electrode was then functionalized with streptavidin by incubating in PBS solution containing 10 μg/mL streptavidin for 60 min at 25 °C and was later rinsed in PBS. The biotylated ssDNA aptamer was immobilized on the surface of the modified electrode via biotin-streptavidin interaction for 45 min at 25 °C. For the purpose of detection 1 nM aptamer was immobilized on the modified electrode surface. The PEDOT/AuNP/aptamer modified electrode was incubated with different concentration of 17-β-estradiol prepared in binding buffer (Scheme 1).

## 3. Results and Discussion

### 3.1. Characterization of Gold Nanoparticles

**Figure 1.**
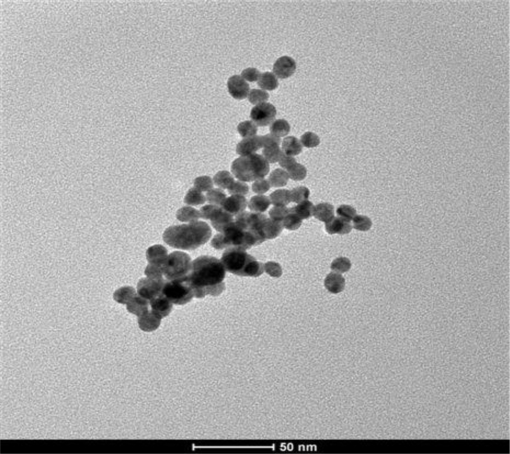
TEM image of gold nanoparticles.

The formation of gold nanoparticles after reduction with citrate was confirmed by the use of UV-visible spectroscopy. A plasmon absorption band characteristic of gold nanoparticles was observed at 525 nm [13,41]. The gold nanoparticles were further characterized with transmission emission microscopy (TEM) to determine the size of the nanoparticles. The nanoparticles have an average diameter of 18 nm. Figure 1 showed the TEM image of the synthesized gold nanoparticles.

### 3.2. Electrochemical Behavior of PEDOT Film Modified Gold Electrode

**Figure 2.**
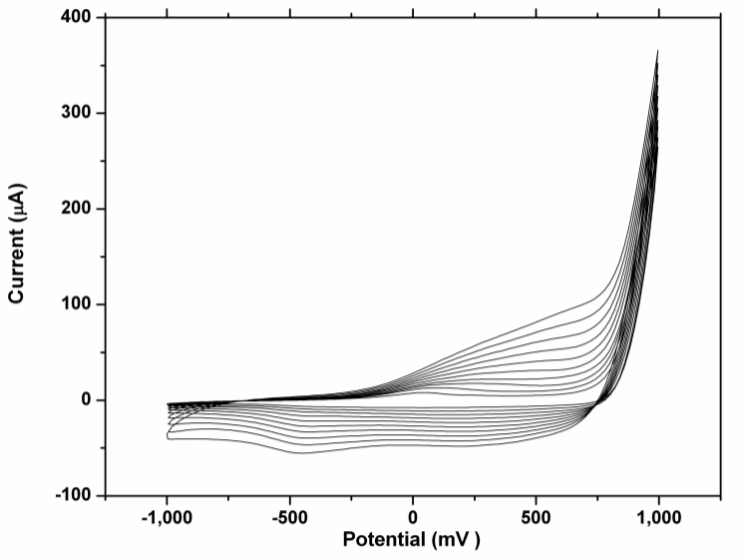
Electropolymerization of 0.1 M 3,4-ethylenedioxythiophene (EDOT) in 0.1 M lithium perchlorate containing 0.1 M sodium dodecyl sulphate.

The PEDOT polymer film was obtained after ten successive cycles in 0.1 M lithium perchlorate containing 0.1 M of EDOT monomer and 0.1 M of sodium dodecyl sulphate (Figure 2).

**Figure 3.**
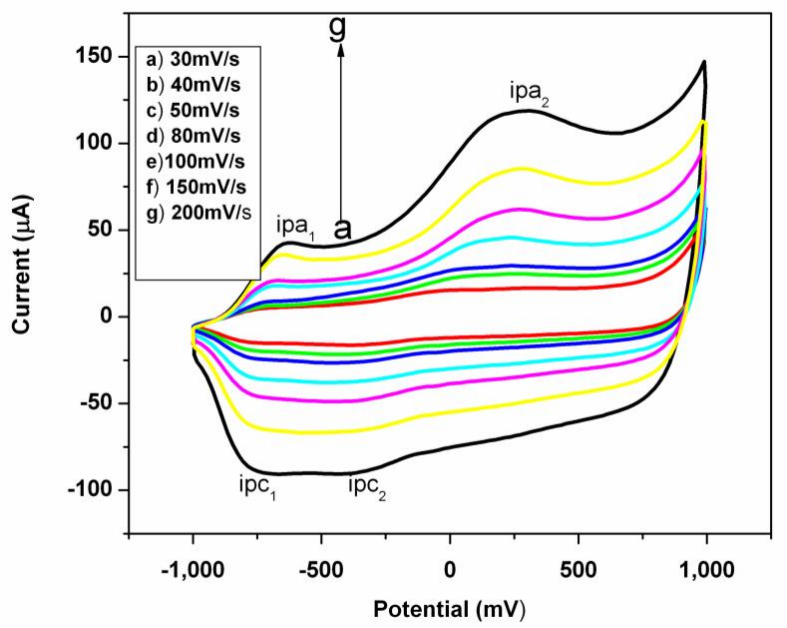
Cyclic voltammograms of PEDOT film modified electrode in 0.1 M lithium perchlorate solution at different scan rates of (a) 30, (b) 40, (c) 50, (d) 80, (e) 100, (f) 150, (e) 200 mV/s.

**Scheme 2.**
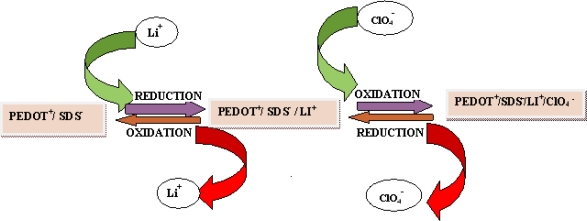
Mechanism of the redox process.

The electrochemical behavior of PEDOT film on a gold electrode resulting from polymerization of EDOT monomer was investigated in 0.1 M lithium perchlorate at different scan rates, by scanning from −1,000 mV to 1,000 mV (Figure 3). PEDOT is electroactive based on the faradaic cathodic and anodic current observed in the CV. However the linear dependence of anodic peak (*i*_pa_) on the scan rate shows that a thin film of surface bound conducting electroactive polymer with a surface confined redox chemistry was obtained [42]. Two sets of peaks were observed with the first redox couple (*i*_pa1_ and *i*_pc1_) situated at about −630 mV while the second anodic peak (*i*_pa2_) was found at about +290 mV. However, cathodic current peak intensity (*i*_pc2_) is not as clearly distinct as (*i*_pa2_) may be because it is located in the vicinity of (*i*_pc1_). It is assumed from the explanation that the existence of the two peak couples is an indication that the mechanism of the redox chemistry may involve both the cation and anion of the electrolytes diffusing in and out of the film. The electrochemical behavior of PEDOT was similar to that obtained in the literature.[43]. The redox chemistry is represented in Scheme 2. The film retained its electroactivity with no shift in the cathodic and anodic peak potential after thirty (30) cycles in 0.1 M lithium perchlorate. This suggest that the film is stable and not falling off the electrode [42,43].

### 3.3. Electrochemical Characteristics of the Nanocomposite

**Figure 4.**
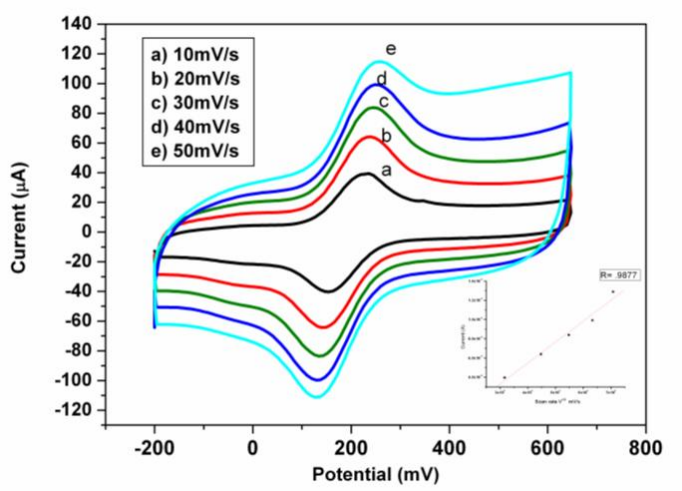
Cyclic voltammograms of the nanocomposite consisting of the conducting polymer PEDOT and gold nanoparticle in 5 mM [K_4_Fe(CN)_6_]/[K_3_Fe(CN)_6_] containing 0.1 M KCl at different scan rates showing electroactivity of the nanocomposite.

The electrochemical behavior of the nanocomposite was investigated using cyclic voltammetry. CV of the nanocomposite consisting of PEDOT and gold nanoparticles (Au/PEDOT/AuNPs) at different scan rates in [Fe(CN)_6_]^−3/−4^ as a redox probe, showed an increase in the peak current as the scan rate increases with no shift in the peak potential (Figure 4).

A linear dependence of anodic current on the scan rate was observed when a plot of *i*_pa_ *versus* scan rate was plotted with a correlation coefficient of 0.987. It can thus be deduced that the platform was characteristic of surface adsorbed species because *I*_pa_ *versus* scan rate was linear. There was no shift in potential and the *i*_pa_/*i*_pc_ is unity also showing the stability of the PEDOT/AuNP platform in [Fe (CN)_6_]^−3/−4^. The rate of electron transport (D_e_) was calculated to be 6.50 × 10^−7^ cm^2^ s^−1^ using the Randle Sevcik equation:

(1) $$I_{p}=2.69\times 10^{5}n^{3/2}\;{\mathrm{AD}}^{1/2}v^{1/2}C$$

where *I*_p_ = peak current, n = number of electron transfer, A = area of an electrode, D = diffusion coefficient, v = scan rate and C = concentration of bulk solution.

This value is high compared to the 6.46 × 10^−8^ cm^2^ s^−1^ reported for PANI doped with polyvinyl sulphonate (PVS) and 8.68 × 10^−9^ cm^2^ s^−1^ without doping [6] The high value may be attributed to the doping of the PEDOT film with gold nanoparticles to produce a nanocomposite platform which further increased the conductivity of the PEDOT film resulting in a D_e_ value approximately one order of magnitude higher than reported for PANI doped with polyvinyl sulphonate (PVS) [6]. The chemistry involved between the PEDOT and Au nanoparticles is more than just physical adsorption. There is a chemical bonding between the well aligned sulphur of the PEDOT and AuNPs due to the affinity of sulphur for gold [44]. The reproducibility of the nanocomposite was investigated by cyclic voltammetry. The nanocomposite platform exhibited reversible electrochemistry in [Fe (CN)_6_]^−3/−4^ with a formal potential of 223 ± 6 mV for six different measurements, demonstrating the good reproducibility of the nanocomposite.

### 3.4. Electrochemical Behavior of Modified Electrode Surface

**Figure 5.**
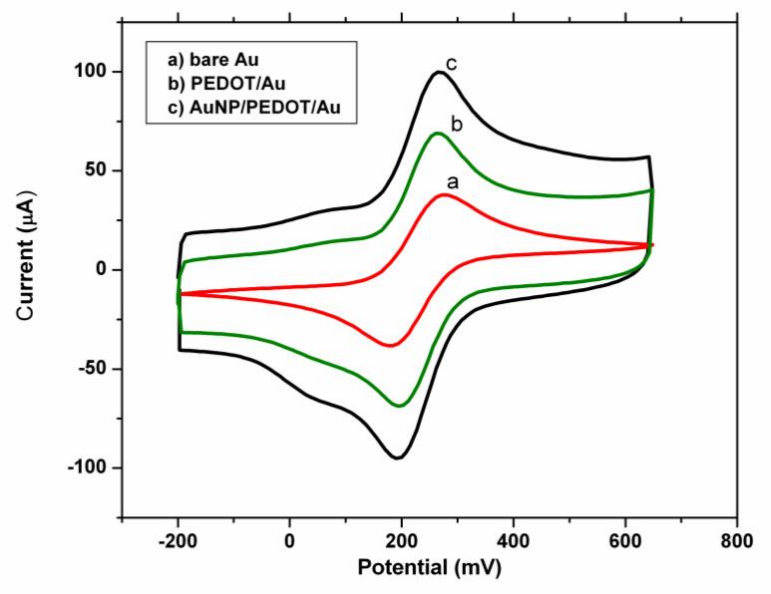
Cyclic voltammograms of 5 mM [K_4_Fe(CN)_6_]/[K_3_Fe(CN)_6_] (1:1) containing 0.1 M KCl at (a) bare Au, (b) Au/PEDOT (c) Au/PEDOT/AuNP at a scan rate of 100 mV/s.

Cyclic voltammograms at the bare gold electrode and the modified electrode in 5 mM [Fe(CN)_6_]^−3/−4^ are shown in Figure 5. A couple of well-defined redox peaks was observed at bare electrode with distinct cathodic peak (*i*_pc_) and the anodic peak current (*i*_pa_) values and a peak to peak separation Δ*E*_p_ of 93 mV. The peak current at Au/PEDOT increased compared to bare electrode, which may be attributed to large surface area and good conductivity exhibited by the PEDOT. The PEDOT film act as a tiny conductive center which facilitate electron transfer which allow more of the [Fe(CN)_6_]^−3/−4^ to be accumulated on the surface of the PEDOT film modified electrode. Au/PEDOT/AuNP exhibited a couple of redox peaks with high peak current and a peak to peak separation Δ*E*_p_ of 73 mV of shown in Table 1 which is an indication that the nanocomposite modified electrode possesses a larger effective surface area as well as good electrical conductivity [42].

### 3.5. Electrochemical Impedance Spectroscopy (EIS) of *Au/PEDOT/AuNP*

**Figure 6.**
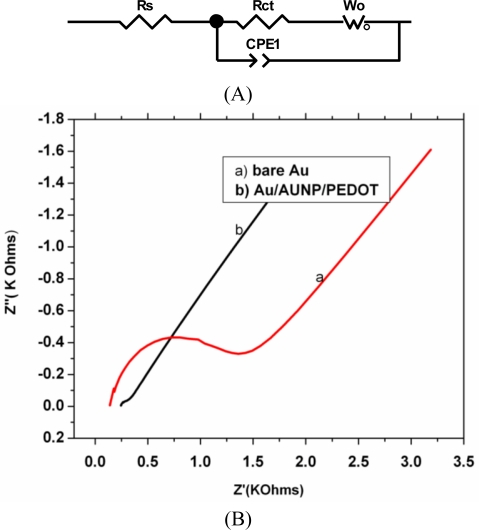
**(A)** Randles equivalent circuit and **(B)** Nyquist plots obtained for (a) bare Au, (b) Au/PEDOT/AuNP modified electrode. EIS measurements were carried out in 5 mM [Fe(CN)_6_]^−3/−4^ containing 0.1 M KCl.

Electrochemical impedance spectroscopy (EIS) has been proven to be one of the most powerful tools for probing the features of surface-modified electrodes. EIS measurements were performed in the presence of 5 mM [K_4_Fe(CN)_6_]/[K_3_Fe(CN)_6_] (1:1) as a redox probe in 0.1 M phosphate buffer containing 0.1 M KCl at a potential of 234 mV and the frequency range is from 0.1 to 10^5^ Hz; the amplitude of the alternate voltage was 10 mV. The resistive and capacitive behavior of an electrode can be quantitatively evaluated by modeling an appropriate Randles equivalent circuit [45]. As shown in Figure 6(A) a Randles modified equivalent circuit was used to fit the impedance data. The parameters of the equivalent circuit included the solution resistance (*R*_s_), the Warburg impedance (*Z*_w_) resulting from the diffusion of the redox probe and charge transfer resistance (*R*_ct_). *R*_ct_ and CPE represent interfacial properties of the electrode which are highly sensitive to surface modification [46,47]. The double layer capacitance (*C*_dl_) is substituted with constant phase element (CPE) when taking into account the electrode roughness. The modification of the electrode with the nanocomposite changes the capacitance and the interfacial electron transfer resistance of the electrode. In the Nyquist diagram the semi circle observed at higher frequency, corresponds to the electron limited process whereas the linear part is the characteristics of the lower frequency range and represents the diffusion limited electron transfer process. The impedance response of the modified electrode in the presence of [Fe(CN)_6_]^−3/−4^ as a redox probe is depicted in Figure 6, while the bare electrode exhibited a very large semicircle at high frequency which represent the R_ct_ with a value of 1.199 × 10^3^ Ω. After the gold nanoparticles and PEDOT film were assembled on the gold electrode the R_ct_ decreased by an order of 1 to give a value of 1.19 × 10^2^ Ω. The decrease was found to be 90% compared to the bare electrode and is attributed to the presence of the AuNP and PEDOT film that plays an important role in accelerating the transfer of electrons, suggesting improved conductivity of the platform. Therefore the platform PEDOT/AuNP can be said to have catalytic effect on the rate of the charge transfer kinetic or the faradaic process of the redox probe. Exchange current was also used as a measure of the rate of electron transfer on the bare and the modified gold electrode. The *i*_0_ of electrode system is given by:

(2) $$i_{0}=\frac{\mathrm{RT}}{{\mathrm{nFR}}_{\text{ct}}}$$

where *R*, *F* and *n* are gas constant, Faraday constant and number of electron transferred, respectively. The *i*_0_ values for the electron transferred reaction of [Fe(CN)_6_]^−3/−4^ on bare gold and Au/PEDOT/AuNP are 2.14 × 10^−5^ A and 2.16 × 10^−4^ A, respectively. From the calculated value it may be deduced that the rate of electron transfer was faster on the PEDOT/AuNP platform than the bare gold electrode. The catalytic effect of this platform may be attributed to enhanced surface and conductivity of the AuNP as well as an increase in the influx of the [Fe(CN)_6_]^−3/−4^ to the surface of the electrode as a result of the electrostatic attraction between cationic platform and the anionic [Fe(CN)_6_]^−3/−4^. To further confirm the catalytic effect of this nanocomposite platform the estimation of the time constant and heterogeneous rate constant was done. The time constant value showed that the faradaic process of the [Fe(CN)_6_]^−3/−4^ probe is one order of magnitude faster on PEDOT/AuNP modified gold electrode than the bare gold electrode as shown in Table 2. In addition the increase in the heterogeneous rate constant at the nanocomposite modified electrode revealed a fast electron transfer of the redox probe due to the catalytic behavior of the nanocomposite platform. This result complemented the result obtained in Figure 4. The Nyquist plot for the nanocomposite is almost a straight line, which is a characteristic of diffusion controlled electrode process.

The electrode coverage is a key factor which can be used to estimate the surface state of the electrode, and the charge resistance is related to it. The surface coverage (*θ*) of the nanocomposite on bare gold electrode can be estimated from EIS according to Equation (3) [48]:

(3) $$\theta =1-\frac{R_{\text{ct}}{}^{\text{modified electrode}}}{R_{\text{ct}}{}^{\text{Bare electrode}}}$$

where *R*_ct_ denotes the charge transfer resistance. The surface coverage of (*θ*) of the electrode was estimated to be 90.1% which will enable more of the bioreceptor (aptamer) to be adsorbed on the surface of the electrode for better sensitivity of the aptasensor to its target.

### 3.6. Quantitative Analysis of 17β-Estradiol Using Aptamer Immobilized PEDOT/AuNP Modified Electrode

Electrochemical analysis was performed to confirm and optimize the condition for ssDNA aptamer immobilized on the surface of the modified electrode. Various concentrations of ssDNA aptamer (0.5 μM–1.0 μM) were initially mobilized on the modified electrode. The current changes in CV were measured before and after immobilization of ssDNA aptamer on the modified and current drop in CV was observed. The difference in current drop varies with the concentration of the ssDNA aptamer on the modified electrode which may be attributed to biotinylated ssDNA aptamer interference with the electron flow [49]. The decrease in current response with increase in concentration may be due to the electrostatic repulsion that exist between the negatively charge phosphate backbone of the DNA aptamer and the negatively charged hydroxyl ion substituent of the target in aqueous solution, which is one of the subtle substituents for aptamer discrimination within molecule of similar structures in the presence of [Fe(CN)_6_]^−3/−4^ as redox probe. Therefore binding of aptamer to charged small molecule in aqueous solution such as 17β-estradiol to aptamer can affect electron flow combined with diffusion rate in the presence of [Fe(CN)_6_]^−3/−4^ [48]. This also showed that PEDOT/AuNP could be a fine platform for immobilization of ssDNA aptamer.

**Figure 7.**
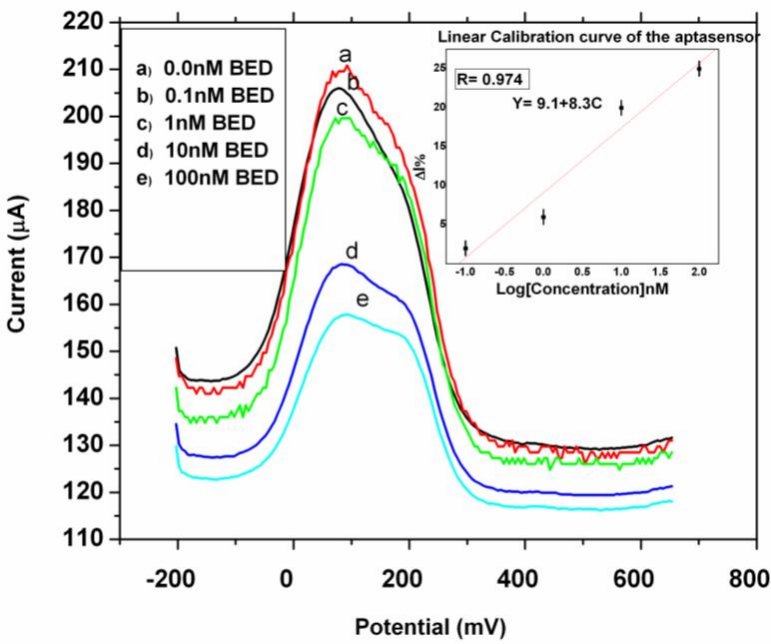
Square wave voltammograms of the electrochemical analysis of 17β-estradiol using 1 nM DNA aptamer immobilized on the nanocomposite platform showing current drop to concentration of target in range of 0.1–100 nM.

The CV and SWV analysis was carried out against a series of 17β-estradiol concentrations (0.1 nM–100 nM) to probe the binding of the 17β-estradiol using 1 nM ssDNA aptamer immobilized on the surface of PEDOT/AuNP modified electrode. The interaction of the target-aptamer complex was monitored by reduction in the electron flux produced from redox reaction between ferrocyanide and ferricyanide. The current drop with increase in concentration was observed in SWV (Figure 7). The decrease in current after 17β-estradiol treatment was based on the specific interaction of aptamer and the target due to electrostatic repulsion that occur between the negatively charged phosphate backbone of the aptamer and negatively charge ion (OH^−^) of the target in solution. The formation of this aptamer-target complex changes the permeability of the layer toward charged ferricyanide ion and consequently the rate of their diffusion [49]. Although a wide range of 17β-estradiol was used, but the minimum concentration with which a current drop occurred was at 0.1 nM 17β-estradiol with slight change in potential and the increasing concentration dependence changes in current was seen till 100 nM in SWV.

The difference in peak potential (Δ*E* = *E*_pa_ − *E*_pc_) from the cyclic voltammograms was increased which was dependent on the concentration of the target molecule. After the target molecule was adsorbed onto the surface of the biosensor, it exhibited a reduced peak current depending on the concentration. The decrease in peak current was observed which could be attributed to slow kinetics of the charge transfer, which is caused by the binding of 17β-estradiol to aptamer [40,49]. However with the series of 17β-estradiol dilutions tested it was found out that 0.1 nM 17β-estradiol was detectable with a lower concentration of 1 nM ssDNA aptamer immobilized on the nanocomposite platform. The high sensitivity of the aptasensor may be attributed to high surface coverage of 90.1% exhibited by the nanocomposite which significantly enhanced the loading of the DNAaptamer probe and hence markedly improve the sensitivity for the target as shown in Table 3 [16].

### 3.7. Specificity and Selectivity Test of the Aptamer

**Figure 8.**
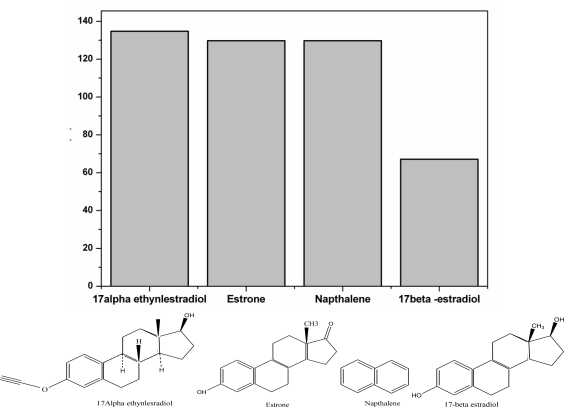
Current response of [Fe(CN)_6_]^−3/−4^ probe in the presence of DNA aptamer to10 nM 17α-ethynlestradiol, estrone, naphthalene and 17β-estradiol respectively.

Beside sensitivity, selectivity is also a remarkable feature for an aptamer sensor. Three different compounds that have similar structures as the target 17β-estradiol were taken as negative control and tested with the aptamer. A different signal must be differentiated from these substances compared to a 17β-estradiol aptamer [49]. Figure 8 compares the response of the fabricated aptasensor to 17β-estradiol and similarly structured substances in the presence of [Fe(CN)_6_]^−3/−4^ as probe. In Figure 8, 10 nM 17α-ethynestradiol, estrone and naphthalene exhibited high current responses when incubated with aptasensor, which may be attributed to low binding action between the aptamer and the analogue chemicals. However, low current response was obtained when 17β-estradiol was incubated with aptasensor due to high binding action that existed between aptamer and 17β-estradiol resulting in the formation of an aptamer-target complex which hindered the electron flow as well as the diffusion rate in the presence of [Fe(CN)_6_]^−3/−4^. The specificity of the target was mostly dependent on change in position or absence of minor functional group such as H or OH in the molecules. This indicates that the designed electrode has a sufficient specificity for 17β-estradiol.

### 3.8. Reproducibility and Stability

The aptasensor is reproducible and stable. Square wave voltammetry repeatedly performed six times by incubating aptasensor with 10 nM 17β-estradiol gave a standard relative deviation of 5.2% (n = 6) confirming the reproducibility of the sensing surface. The stability of the aptasensor was investigated by measuring the response of the aptasensor incubated with 10 nM 17β-estradiol after 15 days. It was found that there was almost 24.5% decrease in peak current response of the aptasensor thus the fabricated aptasensor retained 75.53% of it initial response after it was stored in the refrigerator at 4 °C for 15 days.

## 4. Conclusions

A nanocomposite platform on which a DNA aptamer was immobilized has been developed for the fabrication of an aptasensor employed for the detection of 17β-estradiol, an endocrine disruptor. The aptasensor was found to be sensitive at low concentrations due to the unique properties of the nanocomposite. which allow for the detection of the 17β-estradiol at concentrations as low as 0.02 nM. On the other hand, the result also demonstrated that the PEDOT/AuNP nanocomposite confined on the surface of the gold electrode can provide a promising platform for DNA aptamer construction.

## Figures and Tables

**Table 1.** The electrochemical parameter obtained for the modified surface during CVs.

	***E*_pa_ mV**	***E*_pc_ mV**	***i*_pc_ (A)**	***i*_pa_ (A)**	**Δ*E***	***i*_pa_/*i*_pc_**
BAREGOLD (Au)	273	180	3.788 × 10^−5^	3.693 × 10^−5^	93	0.98
Au/PEDOT	267	191	6.888 × 10^−5^	6.861 × 10^−5^	76	0.99
Au/PEDOT/AuNP	270	197	9.496 × 10^−5^	9.946 × 10^−5^	73	1.05

**Table 2.** Electrochemical impedance kinetic parameters of the bare electrode and the nanocomposite platform.

	**Kinetic parameters**		
	**Exchange current**	**Time constant**	**Heterogeneous rate constant**
Bare electrode	2.14 × 10^−5^ A	1.49 × 10^−3^ s/rad	2.21 × 10^−3^ cm/s
**PEDOT/AuNP** platform	2.16 × 10^−4^ A	9.60 × 10^−5^ s/rad	2.23 × 10^−2^ cm/s

**Table 3.** Comparism of the aptasensor with other sensors.

**Methods**	**Linear range (M)**	**LOD(M)**	**Reference**
Electrochemistry Au/PEDOT/AuNP	0.1 × 10^−9^–100 × 10^−9^	0.02 × 10^−9^	This work
Electrochemistry Bare GCE	4 × 10^−5^–1 × 10^−3^	1 × 10^−5^	[50]
Electrochemistry Poly-serine/GCE	1 × 10^−7^–3 × 10^−5^	2 × 10^−8^	[51]
Electrochemistry GCE/nanoPt-MWNT	5 × 10^−7^–1.5 × 10^−5^	1.8 × 10^−7^	[52]

## References

[1] Stelian L, Ion I, Alina CI (2009). Voltammetric Determination of Phenol at Platinum Electrodes Modified with Polypyrole Doped with Ferricyanide. Rev. Roum. Chim.

[2] Feng Y, Yang T, Zhang W, Jiang C, Jiao K (2008). Enhanced Sensitivity for Deoxyribonucleic Acid Electrochemical Impedance Sensor: Gold Nanoparticle/Polyaniline Nanotube Membranes. Anal. Chim. Acta.

[3] Xiaoxia L, Honglan Q, Lihua S, Qiang G, Chengxiao Z (2008). Electrochemical Aptasensor for the Dteremination of Cocaine Incorporating Gold Nanoparticles Modification. Electroanalysis.

[4] Aixue L, Yang F, Ma Y, Yang XR (2007). Electrochemical Impedance Detection of DNA Hybridization Based on Dendrimer Modified Electrode. Biosens. Bioelectron.

[5] Zhao G-C, Yang X (2010). A Label-Free Electrochemical RNA Aptamer for Selective Detection of Theophylline. Electrochem. Commun.

[6] Mathebe NGR, Morrin A, Iwuoha EI (2004). Electrochemistry and Scanning Electron Microscopy of Polyaniline/Peroxidase-Based Biosensor. Talanta.

[7] Sakmeche N, Bazzaoui EA, Fall M, Aeiyach S, Jouini M, Lacroix JC, Aaron JJ, Lacaze PC (1997). Application of Sodium Dodecyl Sulphate (SDS) Micellar Solution as an Organised Medium for Electropolymerization of Thiopene Derivatives in Water. Synth. Meth.

[8] Ogura K, Nakaoka K, Nakayama M (2000). Studies on Ion Transport During Potential Cycling of a Prussian Blue (Inner) Polyaniline (Outer) Bilayer Electrode by Quartz Crystal Microbalance and Fourier Transform Infrared Reflection Spectroscopy. J. Electroanal. Chem.

[9] Lupu S, Mihailiciuc C, Pigani L, Renato S, Nicolae T, Chiara Z (2002). Electrochemical Preparation and Characterization of Bilayer Film Composed by Prussian Blue and Conducting Polymer. Electrochem. Commun.

[10] Xueliang W, Tao Y, Yuayua F, Kui J, Guiaun L (2009). A Novel Hydrogen Peroxide Biosensor Based on the Synergic Effect of Gold-Platinum Alloy Nanoparticle/Polyaniline Nanotube/Chistosan Nanocomposite Membrane. Electroanalysis.

[11] Yang T, Zhang W, Du M, Jiao K (2008). A PDDA/Poly(2,6-pyridinedicarboxylic acid)-CNTs Composite Film DNA Electrochemical Sensor and its Application for the Detection of Specific Sequences Related to PAT Gene and NOS Gene. Talanta.

[12] Jiang C, Yang T, Jiao K, Gao H (2008). A DNA Electrochemical Sensor with Poly-L-Lysine/Single-Walled Carbon Nanotubes Films and its Application for the Highly Sensitive EIS Detection of PAT Gene Fragment and PCR Amplification of NOS Gene. Electrochim. Acta.

[13] Hussain I, Brust M, Papworth AJ, Cooper AI (2003). Preparation of Acrylate-Stabilized Gold and Silver Hydrosols and Gold-Polymer Composite Films. Langmuir.

[14] Liu F-J, Huang L-M, Wen T-C, Li C-F, Huang S-L, Gopalan A (2008). Platinum Particles Dispersed Polyaniline-Modified Electrodes Containing Sulfonated Polyelectrolyte for Methanol Oxidation. Synth. Meth.

[15] O’Mullane AP, Dale SE, Macpherson JV, Unwin PR (2004). Fabrication and Electrocatalytic Properties of Polyaniline/Pt Nanoparticle Composite. Chem.Commun.

[16] Zhou N, Yang T, Jiang C, Du M, Jiao K (2009). Highly Sensitive Electrochemical Impedance Spectroscopic Detection of DNA Hybridization Based on Aunano-CNT/Pannano Films. Talanta.

[17] Alemán C, Teixeira-Dias B, Zanuy D, Estrany F, Armelin E, del Valle LJ (2009). A Comprehensive Study of the Interactions between DNA and Poly(3,4-ethylenedioxythiophene). Polymer.

[18] Argun AA, Cirpan A, Reynolds JR (2003). Using Poly(3,4-ethylenedioxylthiophene) Polystyrene Sulfonate (PEDO/PSS). Adv. Mater.

[19] Drillet JF, Dittmeyer R, Juttner K (2007). Study of the Activity and Longterm Stability of PEDOT at Platinum Catalyst Support for the DMFC Anode. J. Appl. Electrochem.

[20] Dong-Hun H, Jae-Woo K, Su-Moon P (2006). Electrochemistry of Conducting Polymer 38. Electrodeposited Poly(3,4-ethylenedioxylthiophene) Studied by Current Sensing Atomic Force Micrscopy. J. Phys.Chem. B.

[21] Jui HC, Chi-An D, Wen-Yen C (2008). Synthensis of Highly Conductive EDOT Copolymer Film via Oxidative Chemical *in situ* Polymerization. J. Polym. Sci. A.

[22] Zotti G, Vercelli B, Berlin A (2008). Gold Nanoparticle Linking to Polypyrole and Polythiophene Monolayers and Multilayers. Chem. Mater.

[23] Prakash A, Ouyang J, Lin JL, Yan Y (2006). Polymer Memory Device Based on Conjugated Polymer and Gold Nanoparticles. J Appl Phys.

[24] Pigani L, Heras A, Colina Á, Seeber R, López-Palacios J (2004). Electropolymerisation of 3,4-ethylenedioxythiophene in Aqueous Solutions. Electrochem. Commun.

[25] Xiao YH, Li CM, Toh ML, Xue R (2005). Adenosine 5′ Triophosphate Incorporated Poly(3,4-ethylenedioxythiophene) Modified Electrode a Bioactive Platform with Electroactivity, Stability and Biocompartibility. Chem Biol Interact.

[26] Balamurugan A, Chen S (2009). Silver Nanograin Incorporated PEDOT Modified Electrode for Electrocatalytic Sensing of Hydrogen Peroxide. Electroanalysis.

[27] Zhang Y, Zhou JL (2008). Occurrence and Removal of Endocrine Disrupting Chemicals in Wastewater. Chemosphere.

[28] Schilirò T, Pignata C, Rovere R, Fea E, Gilli G (2009). The Endocrine Disrupting Activity of Surface Waters and of Wastewater Treatment Plant Effluents in Relation to Chlorination. Chemosphere.

[29] Klaus G, Volkmar H, Bjoern T, Einhard K, Hartmut P, Torsen R (2002). Endocrine Disrupting Nonylphenols Are Ubiquitous in Food. Envir. Sci. Technol.

[30] Zhao JL, Ying GG, Wang L, Yang JF, Yang XB, Yang LH, Li X (2009). Determination of Phenolic Endocrine Disrupting Chemicals and Acidic Pharmaceuticals in Surface Water of the Pearl Rivers in South China by Gas Chromatography-Negative Chemical Ionization-Mass Spectrometry. Sci. Total Envir.

[31] Safe S (2004). Endocrine Disruptors and Human Health: Is There a Problem. Toxicology.

[32] Pothitou P, Voutsa D (2008). Endocrine Disrupting Compounds in Municipal and Industrial Wastewater Treatment Plants in Northern Greece. Chemosphere.

[33] Safe S (2005). Clinical Correlate of Environmental Endocrine Disruptors. Trend Endocrine Mat.

[34] Synder SA, Westerhoff P, Yoon Y (2003). Phamaceutical, Personal Care Product and Endocrine Disrupting Chemical in Water Implication for Water Industry. Environ. Eng. Sci.

[35] Jiang JQ, Yin Q, Zhou JL, Pearce P (2005). Occurrence and Treatment Trials of Endocrine Disrupting Chemicals (Edcs) in Wastewaters. Chemosphere.

[36] Amaral Mendes JJ (2002). Endocrine Disrupters a Major Medical Challenge. Food Chem. Toxicol.

[37] Koester CJ, Simoni SC, Esser BK (2003). Environmental Analysis. Anal. Chem.

[38] Cheng AKH, Sen D, Yu HZ (2009). Design and Testing of Aptamer-Based Electrochemical Biosensors for Proteins and Small Molecules. Bioelectrochemistry.

[39] Li X, Shen L, Zhang D, Qi H, Gao Q, Ma F, Zhang C (2008). Electrochemical Impedance Spectroscopy for Study of Aptamer-Thrombin Interfacial Interactions. Biosen. Bioelectron.

[40] Kim YS, Jung HS, Matsuura T, Lee HY, Kawai T, Gu MB (2007). Electrochemical Detection of 17β-estradiol Using DNA Aptamer Immobilized Gold Electrode Chip. Biosens. Bioelectron.

[41] Robertson D, Tiersch B, Kosmella S, Koetz J (2007). Preparation of Crystalline Gold Nanoparticles at the Surface of Mixed Phosphatidylcholine-Ionic Surfactant Vesicles. J. Colloid Interface Sci.

[42] Vasantha VS, Thangamuthu R, Mingchen S (2008). Electrochemical Polymerization of Poly(3,4-ethylenedioxylthiophene) from Aqueous Solution Containing Hydroxyl Propyl-β-cyclodextrine and the Electrocatalytic Behavior of Modified Electrode towards Oxidation of Sulphur Oxoanion and Nitrite. Electroanalysis.

[43] Damlin P, Kvarnström C, Ivaska A (2004). Electrochemical Synthesis and *in situ* Spectroelectrochemical Characterization of Poly(3,4-ethylenedioxythiophene) (PEDOT) in Room Temperature Ionic Liquids. J. Electroanal. Chem.

[44] Shin HC, Su-Moon P (2006). Electrochemistry of Conductive Polymers 39. Contacts between Conducting Polymers and Noble Metal Nanoparticles Studied by Current-Sensing Atomic Force Microscopy. J. Phys. Chem. B.

[45] Abd-Elgawad R, Josep S, Liuis A, Baldrich E, Sullivian C (2005). Reusable Impedimetric Aptasensor. Anal. Chem.

[46] Arotiba OA, Joseph O, Everlyne S, Nicolette H, Tesfaye W, Nazeem J, Baker PGL, Iwuoha EI (2008). An Electrochemical DNA Biosensor Developed on a Nanocomposite Platform of Gold and Poly(propyleneimine) Dendrimer. Sensors.

[47] Arotiba OA, Owino JH, Baker PG, Iwuoha EI (2010). Electrochemical Impedimetry of Electrodeposited Poly(propyleneimine) Dendrimer Monolayer. J. Electroanal. Chem.

[48] Zhang R, Di-Jin G, Chen D, Hu X (2009). Simultaneous Electrochemical Determination of Dopamine, Ascorbic Acid, and Uric Acid Using (Acid Chrome Blue K) Modified Glassy Carbon Electrode. Sens. Acuat. B.

[49] Kim YS, Niazi JH, Gu MB (2009). Specific Detection of Oxytetracycline Using DNA Aptamer-Immobilized Interdigitated Array Electrode Chip. Anal. Chim. Acta.

[50] Salci B, Biryol I (2002). Voltammetric Investigation of β-estradiol. Pharmaceut. Biomed. Anal.

[51] Song JC, Yang J, Hu XM (2008). Electrochemical Determination of Estradiol Using a Poly(L-Serine) Film-Modified Electrode. J. Appl. Electrochem.

[52] Lin XQ, Li YX (2006). A Sensitive Determination of Estrogens with a Pt Nano-Clusters/Multi-Walled Carbon Nanotubes Modified Glassy Carbon Electrode. Biosens. Bioelectron.

