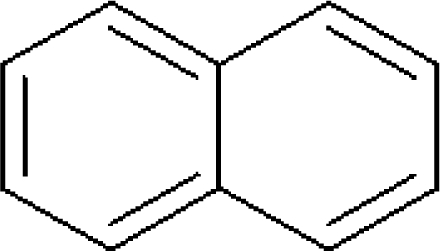# Correction: Baker, P. *et al*. Electrochemical Aptasensor for Endocrine Disrupting 17β-Estradiol Based on a Poly(3,4-ethylenedioxylthiopene)-Gold Nanocomposite Platform. *Sensors* 2010, *10*, 9872–9890

**DOI:** 10.3390/s110202175

**Published:** 2011-02-15

**Authors:** Rasaq A. Olowu, Omotayo Arotiba, Stephen N. Mailu, Tesfaye T. Waryo, Priscilla Baker, Emmanuel Iwuoha

**Affiliations:** Sensor Lab, Department of Chemistry, University of the Western Cape, Bellville, 7535, South Africa; E-Mails: rolowu@uwc.ac.za (R.A.O.); oarotiba@uwc.ac.za (O.A.); 2970836@uwc.ac.za (S.N.M.); twaryo@uwc.ac.za (T.T.W.); eiwuoha@uwc.ac.za (E.I.)

Herewith please find corrected structures for Figure 8 in our paper published in *Sensors* in 2010 [1].

The structure for **17α-ethynylestradiol** used for the analysis is shown below:

**Figure:**
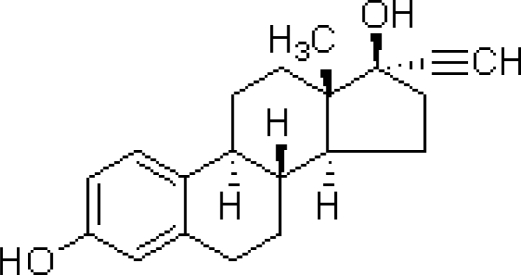

With product number E4876-1G from sigma Aldrich.

The corrected structure of **naphthalene** is below with no double bond in between carbon 2 and 7.

**Figure:**